# Oxytetracycline Degrading Potential of *Lysinibacillus* sp. Strain 3+I Isolated from Poultry Manure

**DOI:** 10.1155/2022/2750009

**Published:** 2022-03-24

**Authors:** Suruttaiyan Sudha, Nivethitha Parthasarathi, Duraisamy Prabha, Palanivel Velmurugan, Subpiramaniyam Sivakumar, Vijayakumar Anitha, Anupama Shrestha, Arunachalam Chinnathambi, Sulaiman Ali Alharb, Perumalsamy Lakshmanaperumalsamy

**Affiliations:** ^1^Department of Environmental Sciences, Bharathiar University, Coimbatore, Tamil Nadu, India; ^2^Centre for Materials Engineering and Regenerative Medicine, Bharath Institute of Higher Education and Research, Selaiyur, Chennai 600073, Tamil Nadu, India; ^3^Department of Bioenvironmental Energy, College of Natural Resources and Life Science, Pusan National University, Miryang-Si, Gyeongsangnam-do 50463, Republic of Korea; ^4^Department of Plant Protection, Himalayan College of Agricultural Sciences and Technology, P.O. Box 44600 Kalanki, Kathmandu, Nepal; ^5^Research Institute of Agriculture and Applied Science, 2356 Tokha, Kathmandu, Nepal; ^6^Department of Botany and Microbiology Department, College of Pharmacy, King Saud University, Riyadh 11451, Saudi Arabia

## Abstract

Oxytetracycline (OTC) which is a broad-spectrum veterinary tetracycline antibiotic is extensively used in poultry farms as a prophylactic, therapeutic, and growth stimulator. Upon administration, unmetabolized OTC is excreted from the animal body through droppings and accumulated in litter in the poultry industry. This study aimed at investigating the OTC degradation potential of an-OTC tolerant bacterial strain, isolated from poultry manure. The isolated strain's morphology, biochemical properties, and 16S ribosomal RNA (rRNA) gene sequence confirmed that it belonged to the *Lysinibacillus* genus. To measure the residual OTC concentration, a high-performance liquid chromatography method was used. OTC degradation rates were 2.579 mg L^−1^d^−1^ with *Lysinibacillus* strain 3+I and 1.149 mg L^−1^d^−1^ without *Lysinibacillus* strain 3+I. In the presence of strain 3+I, the half-life significantly reduced to 2.68 days, compared to 6.03 days without strain 3+I. The strain demonstrated 85% removal with the OTC concentration of 10 *μ*g/ml. The influence of pH, temperature, carbon sources, and nitrogen source, which influence degradation, were also investigated. The optimum condition favouring degradation was pH 6 at a temperature of 30°C. In addition, *Lysinibacillus* sp. strain 3+I's ability to degrade OTC in poultry litter offers a promising approach to treat poultry manure and effluent containing OTC, preventing its contamination in the environment.

## 1. Introduction

Oxytetracycline (OTC) is a broad-spectrum antibiotic that is used in livestock, particularly poultry, due to its high rate range of application in livestock [1]. For growth promotion and disease prevention, chickens are commonly given OTC through their feed and water. In the poultry industry, oxytetracycline is used to treat diseases such as chronic respiratory disease, infectious coryza, and fowl cholera [2, 3]. Veterinary antibiotics are excreted unchanged either as the parent compound or as their metabolites (epimers or isomers) in urine and feces (70–90%, depending on the species) from the animal's gut because of low absorption and inadequate metabolic rates in the animal's gut after administration [4–6]. Since livestock manures are loaded with antibiotics, they are a major source of OTC, as they are applied to agricultural fields as fertilizers. Antibiotics present in soil have negative effects on the environment because they are difficult to degrade in the environment, even at very low doses, distressing crucial ecological activities facilitated by microbes such as nutrient regeneration, biogeochemical cycles, and contaminant degradation [7]. OTC residues have been found in chicken products, poultry droppings, and chicken by-products [8–12]. Considering that the by-products from poultry droppings are used in the production of feed for other animals and that the poultry manure is used for fertilizing, it is of great relevance that OTC is found in poultry droppings [6, 13] resulting in the contamination of soil and water, thus challenging public and animal health. At low concentrations, OTC and its metabolites increase the development of antibiotic-resistant bacteria (ARB) and accelerate the development, mobilization, and transmission of antibiotic-resistant genes (ARG) [14, 15]. This results in altering the microbial community structure in the environment, consequently affecting human and animal health [16]. ARGs against pathogens are primarily found in soil, surface water, and ground water [17, 18]. OTC has been reported to be present in soils at parts per million levels because of recurrent applications of manure, occasionally exceeding the 100-ppm level [19], thus adversely affecting soil microbial communities [20], thereby triggering the development of antibiotic resistance genes [21–24]. According to reports, Danish soil levels of OTC are between 2.5 and 50 mg·g^−1^ and in pig manure levels are between 33 and 2000 mg·g^−1^ [25]. It is also possible for soil borne antibiotics to be absorbed by various crops and thus enter the food chain [26]. Current measures for the removal of antibiotics include adsorption [27], precipitation [28], irradiation [29], hydrolysis [30], and biodegradation [31]. Composting and anaerobic digestion assisted by the addition of amendments such as sawdust and biochar have also been reported, which speed up the reaction to reduce antibiotics in manures [32, 33]. Comparatively, biodegradation can be viewed as a feasible technology for scavenging OTC from the environment; low cost and simple operation are some of its potential advantages [34]. Most of the reports pertaining to OTC removal by bacteria were assessed for their removal from wastewater facilities. Migliore et al. [35] reported the complete degradation of 100 *μ*g ml^−^1 of OTC by the fungus *Pleurotus ostreatus* SMR684. Corcoran et al. [36] studied the pharmaceuticals that are affecting some of the fish species in the aquatic environment. Moreover, Maki et al. [37] proved as an evidence that tetracycline can be degraded by microbes. The removal of OTC by *B. cereus* has been established by Dharmadasa et al. [38], with 56 percent of OTC elimination in a 14-day timeframe. The ability of the ligninolytic fungi in the degradation of different pharmaceutical compounds such as ibuprofen, citalopram, diclofenac, sulfamethoxazole, carbamazepine, and naproxen has also been reported [39]. The tolerance of *Acinetobacter*, *Stenotrophomonas maltophilia*, and *Aeromonas veronii* to OTC in freshwaters of Ireland has been reported by Ding et al. [40]. A reduction of 78.78% of OTC was demonstrated with the bacterial strain *Ochrobactrum* sp. KSS10 [41]. This study was conducted in view of the facts above in order to determine whether *Lysinibacillus* 3+I from poultry manure could remove OTC from aqueous solutions.

## 2. Materials and Methods

### 2.1. Sample Collection

Poultry manure was collected from the piled-up manures of five poultry farms randomly located at Coimbatore, Tamil Nadu, India. During August 2019, poultry litter per farm per week was collected using a shovel. We collected manure samples and stored them at −20°C in sterile vials. Homogenised samples were used for the experiments.

### 2.2. Chemicals and Media

For this study, Sigma-Aldrich (St. Louis, MO, USA) provided oxytetracycline of high purity (98%). Inorganic salt medium (ISM) was comprised of—(NH4)2SO4 (2.0 g), K_2_HPO_4_ (0.5 g), and NaH_2_PO_4_ (0.5 g) adjusted to pH 7.0. Growth media—nutrient broth and nutrient agar, Mueller–Hinton agar, and OTC (g/l) constituted of (NH4)2SO4 (2.0 g), K_2_HPO_4_ (0.5 g), and NaH_2_PO_4_ (0.5 g). The solid medium contained 20 grams of agar per litre. Hi-Media provided commercial-grade antibiotic susceptibility discs for six antibiotics: tetracycline, gentamicin, ceftriaxone, oxytetracycline, erythromycin, and vancomycin. Merck provided acetonitrile and methanol of high-performance liquid chromatography grade.

### 2.3. Strain Enrichment and Isolation

One gram of poultry manure was transferred to a 250-ml Erlenmeyer flask containing 100 ml of inorganic salt medium (ISM) containing 100 g/ml of OTC. Incubation was carried out at 30°C and 150 rpm/min on a reciprocal shaker for seven days. One ml of this mixture was added to fresh ISM media containing OTC, after every seven days. This was performed four times. The same amount of enrichment culture that exhibited maximum growth with OTC was transferred into sterile test tubes and mixed with 9 ml sterile distilled water, and then serially diluted 10-fold. By using the spread plate technique, 1 ml of the serially diluted culture was inoculated onto OTC-ISM agar plates with 100 *μ*g/ml OTC and incubated at 30°C for 48 hours. Using OTC as the sole carbon source, we isolated colonies based on their ability to grow. Using the OTC-ISM agar medium again, the effectiveness of the growth was confirmed at incubation intervals of 0, 1, 2, 3, 5, 7, 14, and 21st days with 100 *μ*g/ml of OTC in ISM broth at 30°C, pH 7.0 and 150 rpm. The growth of the isolated bacteria was determined with a UV-Visible Spectrophotometer (Shimadzu, UV 1800). We chose to investigate the OTC-degrading efficiency of the strain showing maximum growth.

### 2.4. Strain Identification

According to the bacterial identification manual, the physiological and biochemical properties were determined.

### 2.5. Cell Morphology

Scanning electron microscopy (Quanta 200, Icon Analytical Manufacturer) was employed for the observation of the morphological features of the isolated strain.

### 2.6. Biochemical Identification

A series of tests, which included starch hydrolysis, urease utilization, hydrogen sulphide production, tests for catalase, methyl red and carbohydrate fermentation, indole production, oxidase and citrate utilization, and gelatine liquefaction, were carried out (Bergey's Manual of Systematic Bacteriology).

### 2.7. Maintenance of Pure Culture

The pure cultures were kept at −20°C in nutritional broth with 15% glycerol and maintained in a refrigerator. Continuous subculturing on ISM agar was performed every 10 days to avoid any contamination and other problems.

### 2.8. Molecular Identification

As described in our previous study [42], we performed 16srRNA sequencing analysis following the method of [43]. We extracted the strain's genomic DNA and amplified it using bacterial primers 27 F (5′ AGAGTTTGATCMTGGCTCAG 3′) and 1492 R (5′ TACGGYTACCTTGTTACGACTT 3′) in polymerase chain reaction using the following thermal cycling conditions: 94°C for 3 min, 30 cycles of 30 seconds each at 94°C, 60°C, 72°C, and a terminal step at 72°C for 10 minutes [43]. Montage PCR Clean-up Kit (Millipore) was used to remove unincorporated PCR primers and dNTPs from PCR products. In this case, 27F/1492R primers were used to sequence the PCR product. AmpliTaq® DNA polymerase (FS enzyme) was used in the sequencing reactions (Applied Biosystems) with an ABI PRISM® BigDye^TM^ Terminator Cycle Sequencing Kit. In the National Centre of Biotechnology Information (NCBI), the results of the nucleotide sequence analysis were submitted to BLAST analysis (https://blast.ncbi.nlm.nih.gov) in order to generate a universal accession number. Molecular Evolutionary Genetics Analysis Tool (MEGA-X) was used to construct a phylogenetic tree using query and control sequences.

### 2.9. Phylogenetic Tree Construction

Utilizing MEGA-X (Molecular Evolutionary Genetics Analysis Tool), a phylogenetic tree was constructed from queries and control sequences by the neighbor-joining method, which is based on the Jukes–Cantor model. A neighbor-joining method was used to infer the evolutionary history [44]. Bootstrap consensus trees were derived using 1000 replicates [45]. In this case, it is interpreted as representing the evolutionary history of the taxa examined [45]. A partition is collapsed if it has been replicated in fewer than 50% of bootstrap replications. On the branches of the trees, the percentage of replica trees is shown in which the associated taxa clustered together in the bootstrap test (1000 replicates) [45]. A Jukes–Cantor method [46] was used to compute evolutionary distances. Units of the evolutionary distance are the number of base substitutions per site. The analysis involved 11 nucleotide sequences. The partial deletion option was utilized for positions with less than 95% site coverage, thus allowing for fewer than 5% alignment gaps, missing data, and ambiguous bases (partial deletion option). A total of 1257 positions were included in the final dataset. MEGA-X was used to carry out the evolutionary analyses [47].

### 2.10. Susceptibility Tests for Antibiotics

The isolate was subjected to the antibiotic susceptibility test following the disc diffusion method [48, 49] for six different antibiotics, namely, gentamycin, tetracycline, oxytetracycline, vancomycin, erythromycin, and ceftriaxone. Discs of antibiotics were incubated at 30 degrees Celsius for 24 hours on Mueller–Hinton agar plates inoculated with pure cultures of the isolate. The diameter of the zone of inhibition was measured based on Vlkova et al. [50], and the zones were divided into sensitive zones (≥21 mm), intermediate zones (16–20 mm), and resistant zones (≤15 mm).

### 2.11. OTC Degradation in Culture Solution by the Strain 3+I

The potential of the strain 3+I to degrade OTC along with their corresponding cell growth was estimated over a period of 7 days. Timed sampling and dilution counting was employed to calculate OTC removal efficiency and cell growth. To measure growth, we quantified colony-forming units (CFU) on agar plates, and residual OTC concentrations were measured using high-performance liquid chromatography. We developed the isolate's dynamic curve by mixing 100 ml of ISM with 10 g/ml OTC at a pH of 7.0 with 3% precultured bacteria and then inoculated the flasks at 30°C and 150 rpm on a shaker. To evaluate the optimum conditions for the degradation of OTC, experiments were performed by varying the initial concentrations of OTC (1–25 *μ*g/ ml), pH (5–9) temperatures of 20, 30, 35, 40, and 45°C, nitrogen sources such as KNO_3_, NH_4_NO_3_, NH_4_Cl, and NaNO_3,_ and carbon sources including sucrose, glucose, starch, and fructose.

### 2.12. Stock and Standard OTC Solutions

Dissolve 10 mg of OTC in 10 mL of HPLC-grade water to make OTC stock solutions of 1 mg/ml. Amber glass vials were used to prevent the photodegradation of stock solutions. We diluted the stock to produce 1, 2, 3, 10, and 50 *μ*g/ml concentrations for the calibration curve.

### 2.13. Mobile Phases and Preparation of Samples

Acetonitrile and oxalic acid (10 mM) in the ratio 9 : 1 and 2 : 3 ratios were employed as mobile phases to elute OTC. The mobile phases were sonicated for 5 minutes prior to use. For HPLC determination, aliquots of 0.5 ml were taken from the broth culture on days 0, 1, 3, 5, and 7, and transferred to a sterile 1.5-mL microcentrifuge tube. 0.5 mL extracting solvent comprised of 1 N HCl + 100% acetone + 2 g/L Na_2_EDTA, was added to the tube, vortexed thoroughly, and subject to 15-minute sonication. This mixture was then centrifuged for 1 minute at 14000 rpm. The supernatants collected with a syringe were filtered through 0.45-m hydrophilic PTFE Millipore Millex syringe filter [51] into a sterile microcentrifuge tube. The experiments were performed in triplicate. Percent degradation was calculated as follows:

(1) $$\begin{array}{c} \text{degradation efficiency}\left(\%\right)=\frac{C_{\text{initial}}-C_{\text{residual}}}{C_{\text{initial}}}, \end{array}$$

where *C* denotes the OTC concentration in the culture solution.

### 2.1.4. Statistical Analysis

This study used Microsoft Office Excel 2010 (Microsoft, Inc., Redmond, WA, USA) for data processing. The error bars in the figures indicate the mean and standard deviation (mean *∗* SD) of the results (*n* = 3). An unpaired *t*-test was used to analyze changes in the rate of OTC degradation with and without *Lysinibacillus* sp. 3+I. DMRT was performed with SPSS (version 25) to optimize parameters based on the data.

## 3. Results

### 3.1. Isolation and Identification of OTC Degrading Strains

**Figure 1:**
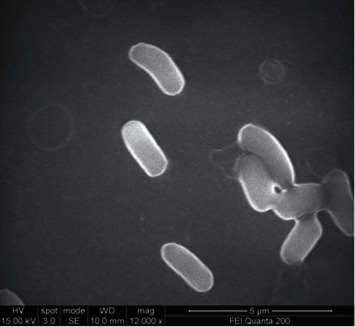
SEM image of *Lysinibacillus* sp. strain 3+I.

We isolated ten different bacterial colony types from poultry manure and grew them on 100 *μ*g/ml OTC agar medium. The isolate OTC-6 showed a higher growth (8.46 ± 0.02 log CFU/ml) with OTC and hence selected for further study. This isolate was labelled as 3+I for experimental purposes. The isolated strain was identified as rod-shaped, Gram-positive bacteria that could form spores and was creamy white in colour as observed from the morphological and biochemical analysis. The scanning electron micrograph (SEM) of *Lysinibacillus* 3+I revealed the cells as rod-shaped and smooth (Figure 1). The biochemical tests showed the isolate to be positive for lactose, sucrose, glucose, starch hydrolysis, catalase, methyl red, and H_2_S production and negative for urease, indole utilization, citrate, gelatin, and oxidase.

### 3.2. Molecular Characterization of Strain 3+I

**Figure 2:**
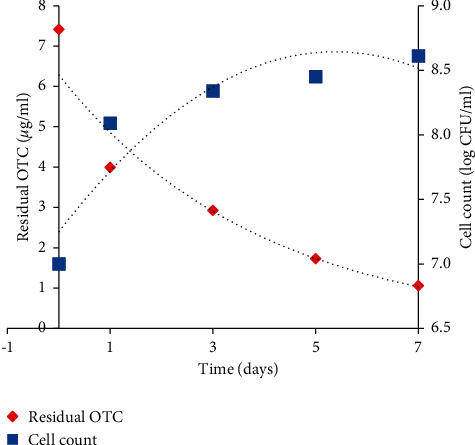
Growth curve and degradation dynamics exhibited by *Lysinibacillus* sp. 3+I strain for 10 *μ*g/ml of OTC.

Both strands of the 16S rRNA gene sequence of the isolate were sequenced using PCR techniques using 16S rRNA universal primers (Figure 2). In the National Centre of Biotechnology Information (NCBI), the resulting nucleotide sequences were submitted to BLAST analysis (https://blast.ncbi.nlm.nih.gov) and deposited in the GenBank database (MN435620) for universal accession. Using Molecular Evolutionary Genetics Analysis Tool (MEGA-X), a phylogenetic tree was constructed using query and control sequences. BLAST analysis of the 16S rRNA sequence of the isolate exhibited close similarity to the genus *Lysinibacillus*, based on its biochemical and morphological characteristics.

### 3.3. Susceptibility Tests for Antibiotics

The susceptibility tests performed using various antibiotics such as gentamicin, tetracycline, erythromycin, ceftriaxone, vancomycin, and oxytetracycline showed resistance of the isolate 3+I to the studied antibiotics. The zone of inhibition was measured to be 7.6, 8.2, 7.9, 7.3, 7.6, and 8.7, respectively. In accordance with Vlkova et al. [50], zones of inhibition exhibited by the isolate were less than <15 mm implying that the isolate was resistant to the tested antibiotics.

### 3.4. OTC Removal by Strain 3+I

**Figure 3:**
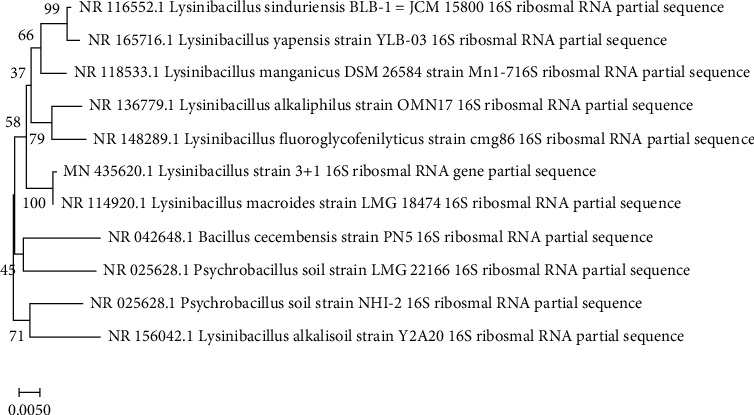
This phylogenetic tree shows the relationship between *Lysinibacillus* species according to the neighbor-joining method.

OTC removal efficiency and growth of strain 3+I were evaluated for a period of 7 days with 10 *μ*g/ml of OTC as initial concentration. The cell count increased with an increase in the incubation time, ranging from 7.0 to 8.61 log CFU/ml (Figure 3), with a reduction in the OTC concentration in the culture solution. Strain 3+I exhibited a significant degradation of 85% over a period of 7 days. OTC concentration was determined by calculating the difference in concentrations between the first (1st) and the final (7th) days of incubation.

### 3.5. Biodegradation Potential of Strain 3+I

Using first-order kinetics, the degradation data showed a significant reduction in the OTC concentration with increasing days, with an *R*_2_ of 0.97. With strain 3+I in the medium, the degradation rate was found to be 2.579 mg/L/d, which is considerably higher than 1.149 mg/L/d in the abiotic control medium (without strain 3+I), denoting that strain *Lysinibacillus* sp. 3+I had an impact on the reduction of OTC from the medium. In the presence of strain 3+I, 2.68 days was recorded as half-life for OTC in the medium, which is significantly less than the control of 6.03 days suggesting such that microbial degradation is superior to abiotic processes.

**Figure 4:**
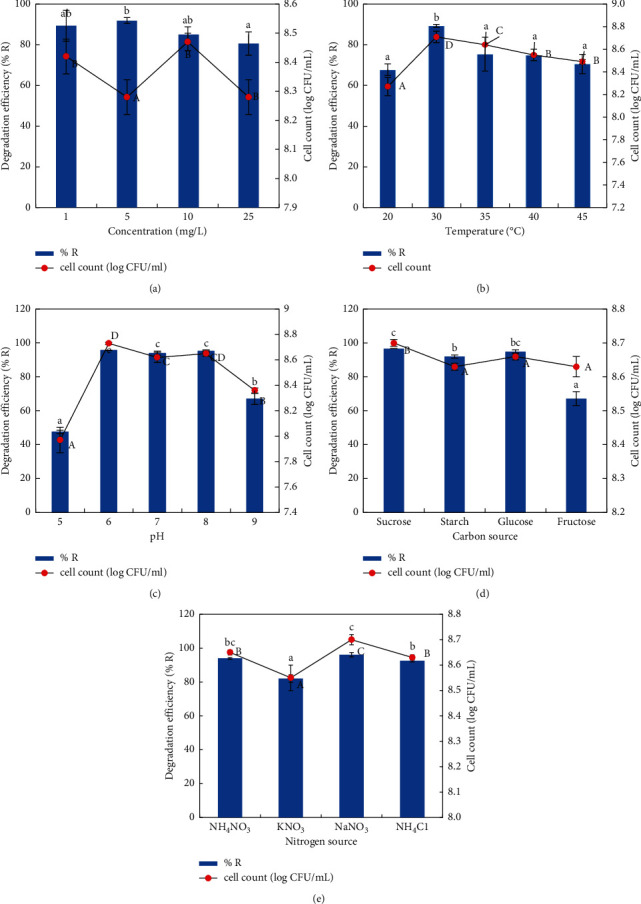
Influence of various parameters on OTC degradation (%) by strain 3+I. (a) Initial concentration of OTC, (b) temperature, (c) pH, (d) nitrogen sources, and (e) carbon sources. In each bar plot, different alphabets in small letters show significant (*P* 0.05) variations (*P* < 0.05) in % R between treatments, and capital letters show significant (*P* 0.05) variations (*P* < 0.05) in the cell count between treatments (SPSS 25, DMRT).

**Figure 5:**
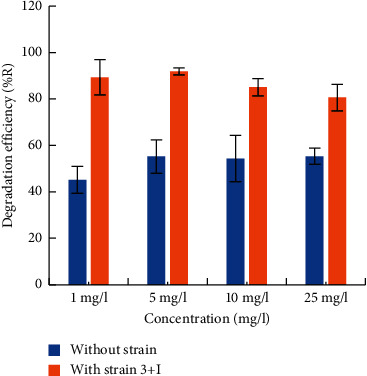
OTC removal with and without the inoculation of *Lysinibacillus* 3+I.

To find the best parameters for boosting OTC removal, the elimination of OTC and cell development of strain 3+I under a variety of culture conditions, including variation in temperature, initial OTC concentrations, pH, carbon, and nitrogen sources were studied. Removal efficacy of OTC at the end of 7 days at 30°C was 89, 92, 85, and 81 percent, respectively, for OTC concentrations of 1, 5, 10, and 25 *μ*g/ml, and the degradation efficiency was highest (92 percent) with 5 *μ*g/ml OTC concentration. The cell growth recorded was higher at 10 *μ*g/ml recording 8.47 CFU/ml and was lower at 5 *μ*g/ml, which was 8.28 CFU/ml (Figure 4(a)). As observed from the results, 10 *μ*g/ml of OTC concentration would have reduced the effectiveness of the organism when compared to 5 *μ*g/ml. This may be attributed to the low bioactivity of the organism under high OTC stress. A similar concept has been explained by Wu et al. [52]. Hence, higher removal percentage would have been observed when the OTC concentration was 5 *μ*g/ml. At lower substrate concentration (5 *μ*g/ml), bacterial cells utilize the substrate at a breakaway time. When the concentration of the substrate is depleted (>90%), the bacterial cells die due to the non-availability of substrate. Hence, the viable cell count (CFU) is less on the 7th day. At a higher concentration, the substrate is still available on the 7th day, and hence, the number of viable cells (CFU) is at higher level. The death of bacterial cells has not commenced yet. If the incubation is prolonged further and the degradation reaches >90%, the growth of the organism would have gradually declined due to want of substrate and the number of CFU will come down. When the temperature was raised from 20°C to 45°C, the average degradation was 68, 89, 75, 75, and 70%, respectively (Figure 4(b)). During the reaction time, the OTC degradation first increased (20–35°C) and then dropped (35–40°C) as the temperature increased. Maximum degradation was observed at 30°C (89%). Moreover, as the pH increased from 5 to 9, the average OTC degradations of 48, 96, 94, 95, and 67% were achieved within 7 days, respectively. Maximum removal (96%) was facilitated when the pH of the culture medium was 6 (Figure 4(c)). When additional carbon sources such as sucrose, glucose, starch, and fructose were supplemented in the growth medium, the degradation was improved only to a maximum of 14% with sucrose. The degradation efficiencies recorded for sucrose, starch, glucose, and fructose were 97, 92, 95, and 67%, respectively (Figure 4(d)). Similarly, nitrogen sources added to the growth medium such as KNO_3_, NH_4_NO_3_, NH_4_Cl, and NaNO_3_ resulted in 82, 94, 93, and 96% degradation efficiencies, respectively (Figure 4(e)). With the addition of nitrogen sources, the degradation was improved maximum only by 13%. These results suggested that strain 3+I does not require additional nitrogen supplements to degrade OTC. Abiotic control samples without the inoculation of strain 3+I were performed in parallel to compute the OTC degradation by abiotic factors. The abiotic control growth medium consisted of sterile ISM media spiked with 10 *μ*g/ml OTC, without the inoculation of strain 3+I. Experimental conditions were similar as in the biodegradation experiments with the strain 3+I, and the experiments were performed in triplicate. OTC degradation percentage was significantly high in the inoculated cultures (85%) when compared to the abiotic control (54%) (Figure 5) indicating 36% higher degradation efficiency of strain 3+I than the abiotic control.

## 4. Discussion

In the present investigation, the strain 3+I that showed good tolerance to OTC was isolated out of ten bacterial strains from poultry manure. Biochemical characterization revealed that the strain was Gram-negative, motile, rod-shaped bacteria with an ability to form spores and the colony was circular oat meal in colour with smooth edges [53]. The 16S rRNA sequence identification shows that strain 3+I has higher similarity with the genus *Lysinibacillus*. In seven days, strain 3+I was capable of removing 85 percent of OTC, according to the findings. The growth curve and degradation dynamics demonstrated a negative correlation, where an increase in cell growth and time resulted in a drop in the OTC concentration. A positive correlation existed between cell biomass and degradation efficiency. Our results are in par with the findings of Li et al. [16], which reported the degradation of 17*β*-estradiol by strain E2S. Several species of bacteria have been reported to be tolerant to OTC—*Acinetobacter*, *Stenotrophomonas maltophilia,* and *Aeromonas veronii* [40], and flavobacterium strains [37] which showed 50% removal of OTC. Harrabi et al. [54] have reported the potential of microbial communities derived from the estuarine regions to degrade OTC. Shi et al. [55] reported OTC degradation by *A*. *nicotianae* strain OTC-16 from aqueous media as well as from manure samples. Abiotic control experiments in the absence of strain 3+I showed 54% removal of OTC, which was significantly less when compared to the experiments in the presence of strain 3+I (85%). As reported by Shi et al. [55] and Harrabi et al. [54], hydrolysis may have contributed to OTC degradation in an aqueous environment, but combined degradation due to hydrolysis and microbial degradation resulted in an increase in OTC removal overall. According to Harrabi et al. [54], microbial communities isolated from estuarine sediments are capable of removing OTC despite the influence of abiotic factors. Enhanced degradation by strain 3+I as indicated by the reduction in half-life (2.68 days) in experiments in the presence of the strain when compared to abiotic control experiments without the strain (6.03 days) suggests that microbial degradation outstands abiotic degradation. With the increase in the concentration of OTC, a gradual decrease in the percent removal was observed. However, removal was low at 1 *μ*g/ml OTC (89%) when compared to 5 *μ*g/ml OTC (92%), which may be because of the less availability of OTC to the organisms as has been reported by Ruan et al. [56]. It has been reported that the decreased degradation at a higher concentration of OTC may be due to the toxicity of OTC at higher or its metabolites in diminishing the degradation ability of the strain [57]. When pH was varied from 5 to 9, the degradation was efficient up to in the pH range 6–8, with the highest removal at pH 6 (96%), whereas it dropped to 66% at pH 9, and the least removal in pH 5 (48%), indicating that the organism could be effective in the wide range of pH 6–8. Lower growth and OTC removal at pH 5 and 9 may be due to the disturbance in the synthesis of enzymes and their associated activities [58]. OTC degradation efficiencies were obtained by maintaining the growth medium at 20°C, 30°C, 35°C, 40°C, and 45°C. At each temperature, 68, 89, 75, 75, and 70% of the OTC was degraded. The average degradation of OTC was first increased and then reduced as the temperature increased from 20°C to 30°C (Figure 4(b)). 30°C (89%) was found to be the ideal temperature for OTC degradation. Microbial processes are affected by the temperature of the growth medium since microorganisms must have a temperature that is conducive to cellular enzyme activity as well as the reaction rate of the cells [41]. *Ochrobactrum* sp. decreased its degradation efficiency as temperature increased, as reported by Shao et al. [41]. As the reaction progressed, KSS10 increased from 20 to 35°C and then decreased from 35 to 40°C. When diverse carbon sources (sucrose, starch, glucose, and fructose) were applied to the growth media, the maximum improvement in removal efficiency by 13% was observed only with sucrose, whereas fructose decreased the removal by 20%, indicating that the strain was able to use OTC as its primary carbon source, thus eliminating the need for additional carbon sources. A similar trend was observed when diverse nitrogen sources were supplemented to the media (ammonium nitrate, potassium nitrate, sodium nitrate, and ammonium chloride), which enhanced removal only by 13% with ammonium chloride.

The ability of bacteria to effectively degrade OTC is said to be associated with enzyme productions such as manganese peroxidase [59], laccase [60], glutathione S-transferases [61], and tetracycline inactivating monooxygenase [62–64]. Hence, the degradation of OTC by *Lysinibacillus* 3+I can be considered to share a similar degradation process. The study shows *Lysinibacillus* 3+I, which is able to utilize OTC for its carbon requirement and operating at pH 6 and 30°C as an appropriate candidate for removing OTC from poultry manure, thus reducing the additional expenditure of adding supplement nutrients to provide optimum conditions.

## 5. Conclusion

To restore our ecosystem and maintain the global carbon cycle established through bioremediation, microorganisms play a critical role. Based on the results of the current study, it is understood that microbes have the ability to clean up contaminants in the environment and could be used as a green instrument for the removal of environmental pollutants. OTC-degrading bacteria have demonstrated their ability to contribute to efforts to mitigate OTC's environmental and public health hazards, and as such, they should be the subject of future research. Our results shows that *Lysinibacillus* 3+I isolated from poultry manure could significantly remove OTC. This organism can be suggested as a suitable candidate for in-depth research and further for incorporation in poultry wastewater pretreatment.

## Data Availability

The data used to support the findings of this study are available from the corresponding author upon request.
